# Visualizing, quantifying, and manipulating mitochondrial DNA *in vivo*

**DOI:** 10.1074/jbc.REV120.015101

**Published:** 2020-12-18

**Authors:** David L. Prole, Patrick F. Chinnery, Nick S. Jones

**Affiliations:** 1 Department of Mathematics, Imperial College London, London, United Kingdom; 2 Medical Research Council Mitochondrial Biology Unit, University of Cambridge, Cambridge, United Kingdom; 3 Department of Clinical Neurosciences, University of Cambridge, Cambridge, United Kingdom

**Keywords:** aging, mitochondrial DNA (mtDNA), mitochondrial disease, mitochondria, microscopy, mitophagy, gene editing

## Abstract

Mitochondrial DNA (mtDNA) encodes proteins and RNAs that support the functions of mitochondria and thereby numerous physiological processes. Mutations of mtDNA can cause mitochondrial diseases and are implicated in aging. The mtDNA within cells is organized into nucleoids within the mitochondrial matrix, but how mtDNA nucleoids are formed and regulated within cells remains incompletely resolved. Visualization of mtDNA within cells is a powerful means by which mechanistic insight can be gained. Manipulation of the amount and sequence of mtDNA within cells is important experimentally and for developing therapeutic interventions to treat mitochondrial disease. This review details recent developments and opportunities for improvements in the experimental tools and techniques that can be used to visualize, quantify, and manipulate the properties of mtDNA within cells.

Mitochondrial DNA (mtDNA) encodes a variety of proteins, peptides, transfer RNAs, and ribosomal RNAs that support the functions of mitochondria and is thereby central to numerous physiological and pathophysiological processes, including development, disease, and aging (1). Both the number of mtDNAs in a cell (mtDNA copy number) and the sequences of mtDNAs are important phenotypic determinants (2). Mutations of mtDNA can cause a spectrum of mitochondrial diseases where the clinical expression depends on the specific mutation and the degree of heteroplasmy (the proportion of mutated mtDNA in a single cell) between WT and mutant mtDNA (3, 4). The copy number of mtDNA is controlled by poorly understood mechanisms but varies between tissues, during aging, and in cancer (5–7). The mtDNA within mitochondria is present within nucleoids (8). Nucleoids contain complexes of mtDNA with proteins and other factors that comprise the machinery required for regulated transcription (8–10). The abundance and sequence of mtDNA can affect mitochondrial function, whereas the mitochondrial network, which is regulated by dynamic fission and fusion events (11), can impact the turnover and copy number of mtDNA (2, 12).

Single-cell studies have shown that mtDNA content and heteroplasmy are dynamic throughout life, with marked heterogeneity (3, 13–15). Although vegetative segregation and relaxed replication of mtDNA appear to be important, it remains unclear how and when these processes are involved in different tissues because most methods for quantifying mtDNA variants are *in vitro* biochemical assays that are destructive to cells and preclude measurements of mtDNA over time (3, 13, 16). Direct visualization of mtDNA *in vivo* can thus offer further mechanistic insight. Visualization of the mtDNA copy number has revealed that mtDNA increases its population during S-phase in the cell cycle (17), that mtDNA copy number differs between tissues and can decline during aging (6), and that mtDNA copy number is reduced in some cancers such as glioma (18). Visualization of mtDNA in yeast has shown that segregation of mtDNA during cell division preserves the density of mtDNA in daughter cells, in part via the semi-regular spacing of nucleoids within mitochondria (19, 20). Visualization of replicating mtDNA nucleoids has revealed that they coincide with endoplasmic reticulum–mitochondria contact sites, mitochondrial fission, and actin (21–23). High-resolution and superresolution microscopy (SRM) imaging has revealed that there are relatively small numbers of mtDNAs per nucleoid (mean ∼1.4, and often only one), that nucleoids have a relatively uniform size of ∼100-nm diameter (23–25), that there are relatively small numbers (∼1–15) of nucleoids per mitochondrion (26), and that mtDNA resides in voids between mitochondrial cristae (27). Fluorescence *in situ* hybridization has shown (in a manner consistent with the low number of mtDNAs per nucleoid) that individual mtDNA nucleoids maintain their genetic autonomy rather than freely exchanging mtDNA between nucleoids (28) and that removal of deleterious mutant mtDNA from the germline may occur after mitochondrial fragmentation (12).

Despite considerable advances in our understanding of mtDNA biology, fundamental questions remain, such as how mtDNA nucleoids are formed and distributed within cells, how mtDNA copy number is controlled, and how mtDNA heteroplasmy is determined in different cells and tissues.

This review aims to assemble the existing suite of experimental tools and techniques that can be used to visualize, quantify, and manipulate mtDNA within cells; it places a particular emphasis on visualization. In the first section, we discuss methods for labeling mtDNA nucleoids in cells. The next section provides details of imaging methods for visualizing mtDNA in cells. Next, we discuss the manipulation of mtDNA in cells. Finally, we discuss some of the future challenges and new approaches in the field that may enable a greater understanding of the roles and regulation of mtDNA in cells. Tools used to probe more general mitochondrial physiology are reviewed elsewhere (29, 30).

## Labeling mtDNA nucleoids in cells

### Desirable properties for tools to label and visualize mtDNA

The experimental tools and techniques that can currently be used to label, visualize, and quantitatively describe the characteristics of mtDNA include those summarized in Table 1. The ideal tool for labeling and visualizing mtDNA would enable the most challenging experimental approaches to investigate mtDNA physiology. These include long-term time-lapse microscopy to monitor mtDNA throughout the life of a cell or organism, superresolution microscopy to determine the architecture of nucleoids and their relationship to mitochondria, and selective visualization of different variants of mtDNA within cells and tissues to reveal the dynamics of each mtDNA variant and their effects on the mitochondria and cells in which they reside. To achieve these aims, the tools for labeling mtDNA would have the following nine challenging but desirable properties. 1) It should selectively label mtDNA rather than nuclear DNA, in both live and fixed cells. 2) It should be nontoxic and nonperturbing, thus allowing visualization over time. 3) It should be photostable for extended periods of video imaging and particle tracking. 4) It should be flexible with respect to spectral characteristics, to enable multicolor imaging with other labels directed to other targets and to enable pulse-chase experiments. 5) It should also be flexible with respect to binding affinity for mtDNA, so that stable or reversible binding can be employed. 6) It should be capable of specifically detecting replicating mtDNA. 7) It should be compatible with SRM to achieve images with the highest spatial resolution. 8) It should be applicable to intravital imaging of tissues and organisms *in vivo*. 9) It should label sequence variants of mtDNA selectively, including single-nucleotide variants, to help understand the pathophysiology of mtDNA heteroplasmy. All of the tools that are currently available have limitations with respect to these desirable properties (Table 1).

**Table 1:** **Tools and techniques for visualizing mtDNA in cells** TIRFM, total internal reflection fluorescence microscopy; CLEM, correlative light and EM; FISH, fluorescence *in situ* hybridization.

Tool/Technique	Advantages	Disadvantages	References
**Microscopy techniques**			
Confocal microscopy	Ease, speed, 3D	Resolution restricted to optical diffraction limit	17
Superresolution microscopy	High resolution	Low number of colors possible (one to three); sometimes restricted modality (*e.g.* TIRFM)	24, 25, 27
Light-sheet microscopy	Speed for large samples, low phototoxicity	Cost of hardware; not maximum resolution	73
EM	Highest resolution	Fixation, heavy metal staining, and sectioning may be necessary	73
Cryotomography	Highest resolution; no fixation, staining or sectioning required	Rapid freezing required	
CLEM	Combination of EM and fluorescence enables correlation of proteins and structures	Expense, technical expertise, laborious	73, 76, 77
**DNA-binding dyes**			
SYBR Green	Live cells	Fixed λ, fixation, and permeabilization disrupt labeling, toxic in some cells, bleaches relatively rapidly	17
SYBR Gold	Live cells	Fixed λ	33
PicoGreen	Live cells	Fixed λ, fixation, and permeabilization disrupt labeling, toxic in some cells, bleaches relatively rapidly	34
EtBr	Live cells in some cases	Fixed UV λ, inhibits mtDNA replication, low membrane permeability, toxic, may require fixation and permeabilization	38
DAPI	Live cells	Fixed UV λ	37
SiR-Hoechst	Live cells, far-red	Fixed λ	43
**Nucleotide analogues**			
EdU-click	Variable λ	Fixation and permeabilization necessary	46
EdU-click with amplification	Variable λ Increased signal/noise ratio	Fixation and permeabilization necessary	17
BrdU	Variable λ	Fixation and permeabilization necessary, harsh denaturing step	45
**Fluorescence-tagged proteins**			
TFAM-FP	Variable λ	Possible perturbing effect of FP tag, overexpression artifacts	53
POLG2-FP	Variable λ, selective for replicating mtDNA	Possible perturbing effect of FP tag, overexpression artifacts	21, 57
**Antibodies**			
Ab-TFAM	Variable λ	Fixation and permeabilization necessary	53
Ab-DNA	Variable λ	Fixation and permeabilization necessary	21
***In situ* hybridization**			
FISH	Variable λ, sequence-specific	Fixation and permeabilization necessary	12, 28
Padlock probes	Variable λ, sequence-specific	Fixation and permeabilization necessary	61, 62
**Targeted nucleases**			
CasPLA	Variable λ, sequence-specific	Fixation and permeabilization required, few NGG PAM sites in mtDNA	63

### Fluorescent DNA-binding dyes

**Figure 1.**
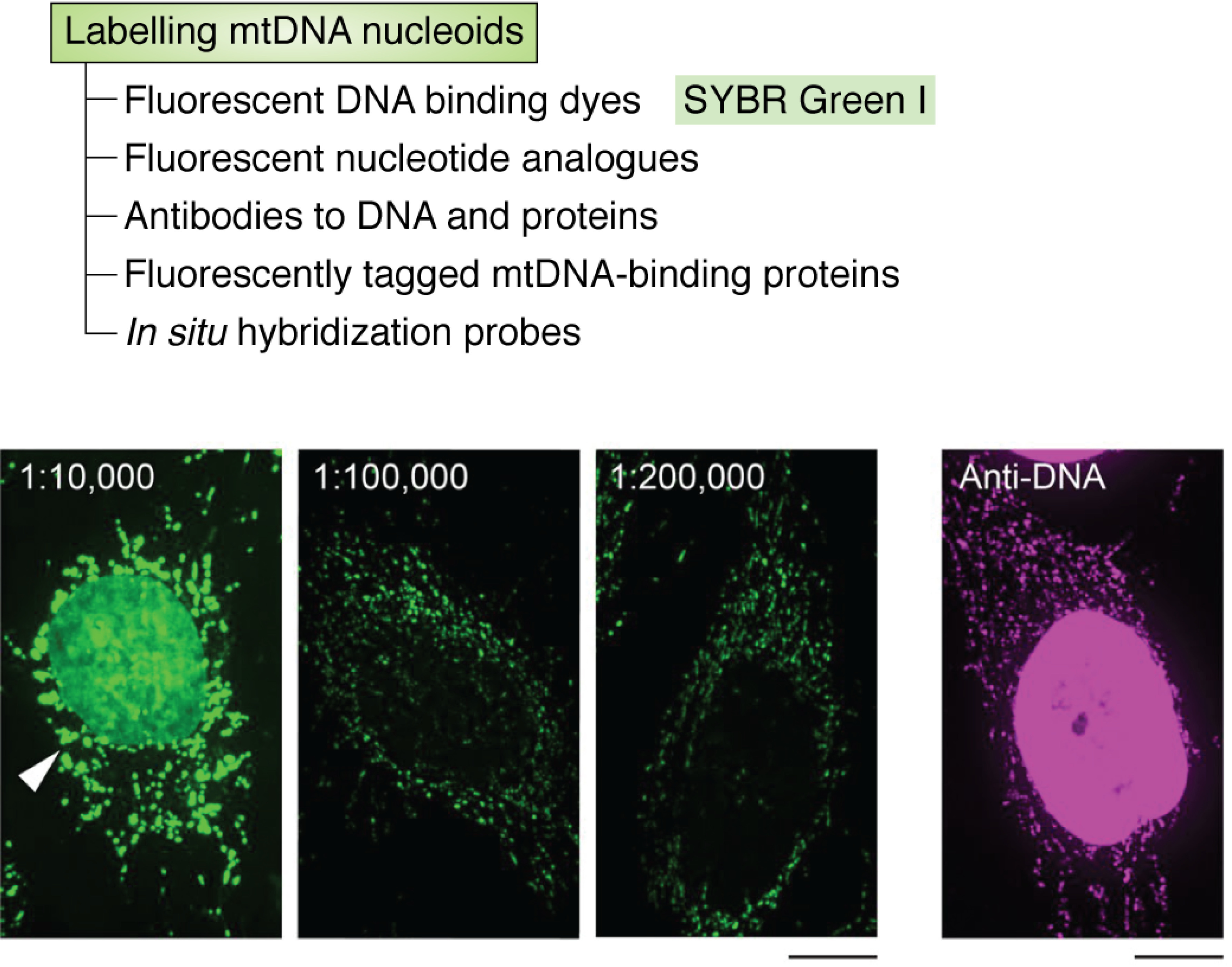
**Labeling mtDNA with SYBR Green I.** Shown is staining of HeLa cells with different concentrations of SYBR Green I. Dilutions of SYBR Green I are shown *above* the images. DNA was immunostained using anti-DNA antibodies in cells without staining with SYBR Green I (*far right panel*). *Scale bars*, 10 μm. The images are taken from previously published data (17); the figure panel has not been changed; and the images are covered by a license (http://creativecommons.org/licenses/by/4.0/).

A wide variety of DNA-binding dyes exist that can label DNA in cells (31, 32), but these have been used mainly to label nuclear DNA. Only a few of these have been demonstrated to label mtDNA within cells. These include SYBR Green I (17, 23), SYBR Gold (33), PicoGreen (21, 34–36), DAPI (37), and ethidium bromide (38). Red fluorescent DNA-binding dyes that label mtDNA have been described recently (39). DNA-binding organic dyes can be relatively bright and photostable and can bind mtDNA stably over periods of days within cells (33). An example of live mammalian cells in which mtDNA nucleoids have been labeled with SYBR Green is shown in Fig. 1 (17).

DNA-binding dyes must traverse the plasma membrane and mitochondrial outer and inner membranes in live cells to gain access to mtDNA in the mitochondrial matrix. The physicochemical properties of dyes have therefore been used to rationalize the choice of dyes that might accumulate in mitochondria and bind to mtDNA, for example yielding SYBR Gold as a suitable dye (33). Relevant properties of the dye include delocalized positive charge to favor uptake across the negative membrane potential of the mitochondrial membrane; lipophilicity, to penetrate the plasma membrane and mitochondrial membranes; high-affinity binding to DNA; and preferably fluorogenicity (*i.e.* a fluorescence enhancement upon binding to mtDNA).

A DNA-binding dye consisting of SYBR Green linked to a cationic rhodamine-B moiety to enable mitochondrial targeting yields green fluorescence in mitochondria but red fluorescence in lysosomes after mitophagy (40). This type of DNA-binding dye/sensor may have utility in revealing mechanisms involved in mtDNA turnover during mitophagy. A ruthenium(II)-peptide conjugate is reported to target mtDNA in live cells and enable the induction of targeted phototoxicity in selected cells (41). The mtDNA can be a target of oxidative attack by hydrogen peroxide and reactive oxygen species. To study this process, a fluorescent mtDNA-tethered peroxide sensor has been reported, comprised of a DNA-binding peptide coupled to a green fluorescent peroxide sensor and a charged red fluorescent dye (42).

The existing organic mtDNA-binding dyes are limited to certain wavelengths of excitation and emission light. SYBR Green, SYBR Gold, and PicoGreen all emit green fluorescence and require illumination in the 488-nm region. EtBr and DAPI require shorter-wavelength illumination in the UV region, which can be biologically damaging. Red fluorescent DNA-binding dyes that label mtDNA have been described recently (39). The lower toxicity of longer-wavelength illumination, together with the expanded spectral flexibility these red dyes provide, may be useful for time-lapse imaging and combination with other fluorescent labels (*e.g.* to identify replicating mtDNA and total mtDNA simultaneously). They may also be suitable for SRM to determine the architecture of nucleoids (39). Other DNA-binding dyes based on Hoechst (43) or rhodamine derivatives (44), that are red fluorescent or far-red fluorescent, have been described recently, but it remains to be determined whether these dyes can be used for visualizing mtDNA.

In addition to labeling mtDNA with a fluorescent DNA-binding dye, it is often experimentally desirable to label other cellular components with antibodies or membrane-impermeant probes, which necessitates fixation and permeabilization of cells. Fixation with paraformaldehyde and detergent permeabilization can reduce staining of cellular mtDNA with DNA-binding dyes (33). Another limitation of current DNA-binding dyes for visualizing mtDNA is their binding to nuclear DNA within the same cells. Extreme dilution of the DNA-binding dye has been demonstrated to minimize this issue by aiding the selective labeling of mitochondrial nucleoids by SYBR Gold (33) and SYBR Green (17) for reasons that remain unclear. DNA-binding dyes also intercalate between the two strands of dsDNA. This may alter the ability of DNA strands to dissociate during replication of both nuclear DNA and mtDNA and may thereby alter cell division and be toxic in some cell types.

### Fluorescent nucleotide analogues

5-bromo-2-deoxyuridine (BrdU) is a nucleotide analog that can incorporate into replicating mtDNA (25, 45). Once incorporated into DNA, the BrdU is inaccessible to antibodies and fluorescent labeling until relatively harsh chemical or enzymatic cleavage of the DNA is used to uncover the BrdU epitope and facilitate binding of labeled antibodies (45).

**Figure 2.**
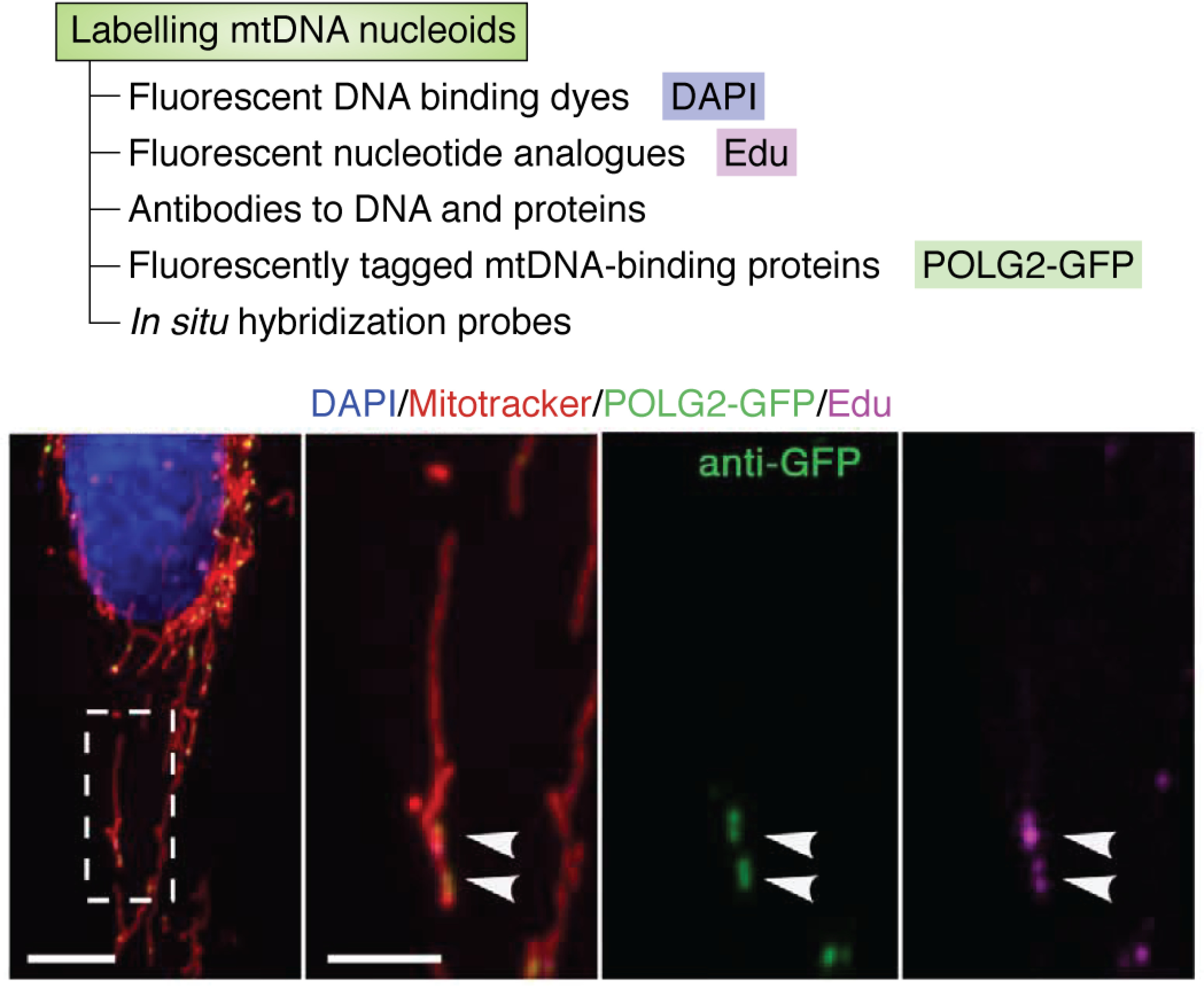
**Labeling replicating mtDNA with EdU and POLG2-GFP.** Shown is a representative U2OS cell expressing POLG2-GFP and labeled with EdU (50 μm), fixed and stained with DAPI (DNA, *blue*), MitoTracker (mitochondria, *red*), anti-GFP–Alexa Fluor 488 conjugate antibody (POLG2-GFP, *green*), and Click-iT EdU-Alexa Fluor 647 (nascent DNA, *magenta*). *Arrowheads*, colocalization. *Scale bar*, 10 μm. The images are reproduced from previously published data (21), reprinted with permission from the American Association for the Advancement of Science.

5-Ethynyl-2′-deoxyuridine (EdU) is another nucleotide analog that can incorporate specifically into replicating mtDNA (17, 46, 47). EdU can be fluorescently labeled using a copper(I)-catalyzed click reaction to form a covalent attachment between the alkyne group of EdU and an azide group on an appropriately conjugated fluorescent dye. Labeling of EdU does not require the denaturation step involved in BrdU labeling, and EdU can be visualized directly (*e.g.* using an azide-Alexa Fluor label) (Fig. 2) (21). Alternatively, a signal amplification step can increase signal/noise ratio by using a fluorescence-conjugated antibody directed against the click-conjugated fluorescent dye to introduce further fluorescent dye molecules (17, 46, 47). For example, an antibody against Oregon Green 488 can amplify the signal from EdU labeled with Oregon Green 488-azide (17).

A wide variety of azide-conjugated fluorescent dyes exist, and the resulting spectral flexibility provides an advantage of EdU and BrdU techniques over currently available organic DNA-binding dyes. Furthermore, a combination of BrdU and EdU can be utilized in sequential pulse-chase experiments, to monitor mtDNA replication (48).

BrdU and EdU incorporate into replicating strands of both nuclear DNA and mtDNA. Specific visualization of mtDNA using these nucleotide analogues may, therefore, require pharmacological inhibition of nuclear DNA replication by drugs, such as aphidicolin (17), or restricted use in postmitotic or quiescent cells. Other limitations are the incorporation of these nucleotides during repair of DNA and the reported cytotoxicity of EdU in some cells (49, 50). Finally, labeling of incorporated EdU and BrdU for most applications requires fixation and permeabilization, as most of the reactive probes are membrane-impermeant; a strategy for labeling EdU and BrdU, or other similar probes, in live cells would be of substantial utility to detect replicating mtDNA. Labeling EdU with a membrane-permeant tetramethylrhodamine azide, together with copper(I) generated *in situ*, has been employed without fixation, but this use has not been developed significantly, in part due to toxicity of the probes used (51).

Halogenated thymidine analogues have also been used to label replicating mtDNA, in a technique termed mitochondrial single-molecule analysis of replicating DNA (52). Relatively short pulses of incubation with the halogenated thymidine derivatives 5-iodo-2′-deoxyuridine or 5-chloro-2′-deoxyuridine can lead to incorporation of these analogues into replicating mtDNA within cells over defined time periods of time (52). Specific antibodies directed against either 5-iodo-2′-deoxyuridine or 5-chloro-2′-deoxyuridine can then be used to visualize the locations of each incorporated analog within single mtDNA genomes, via SRM (52). This technique has revealed mechanisms underlying the replication of mtDNA and the generation of common mtDNA deletion mutants (52).

### Antibodies to DNA and proteins

**Figure 3.**
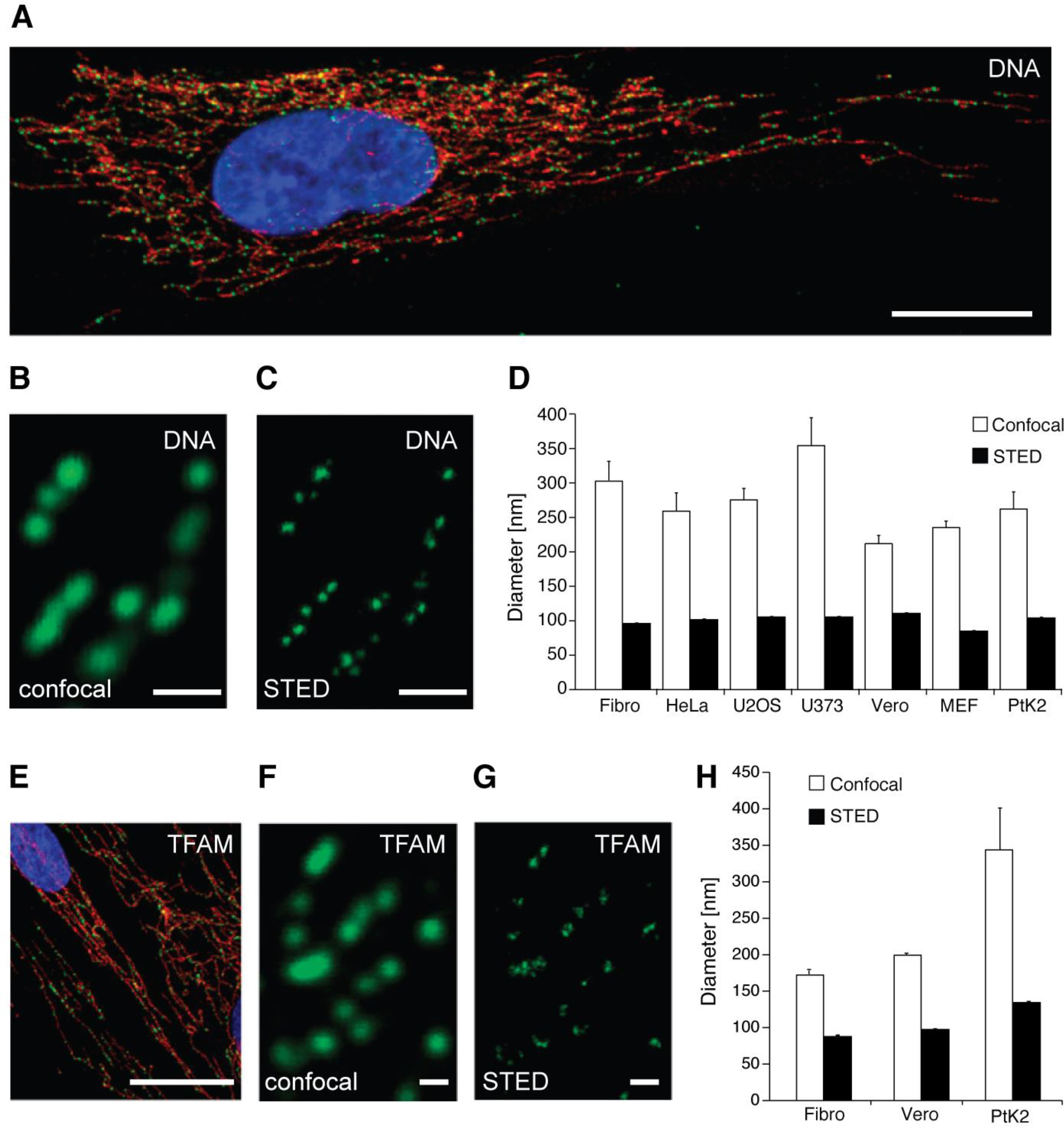
**Labeling mtDNA with antibodies directed against DNA and TFAM.** *A*, human fibroblasts showing mtDNA (*green*, DNA antibodies) localized in nucleoids in the mitochondrial network (*red*, anti-TOM20) with DAPI staining of nucleus in *blue*. *B*, confocal microscopy image of nucleoids labeled with antiserum against DNA. *C*, STED microscopy image of the same nucleoids shown in *B*. *D*, sizes of nucleoids labeled with a DNA antibody as measured using confocal and STED imaging. *E*, TFAM (*green*, anti-TFAM) located in nucleoids within the mitochondrial network (*red*, anti-TOM20) of human fibroblasts. Nuclear DAPI staining is shown in *blue*. *F*, confocal microscopy image of nucleoids labeled with antiserum against TFAM. *G*, STED microscopy image of the same nucleoids shown in *F*. *H*, quantification of sizes of nucleoids labeled with TFAM antibodies, determined by confocal and STED. *Error bars*, S.E. *Scale bars*, 20 μm in *A* and *E* and 0.5 μm in *B*, *C*, *F*, and *G*. The images and figure are reproduced from previously published data (25), reprinted with permission from the United States National Academy of Sciences.

Antibodies raised against DNA can detect mtDNA in fixed, permeabilized cells (10, 23, 25, 47) (Fig. 3). These antibodies do not currently distinguish between nuclear DNA and mtDNA. Antibodies to protein constituents of mitochondrial nucleoids are commonly used to identify mtDNA. A critical component of the mtDNA transcription machinery, and a commonly used marker for nucleoids, is the mitochondrial transcription factor A (TFAM) protein (53–55). An example of fixed, permeabilized cells in which mtDNA nucleoids have been labeled with an antibody directed against TFAM is shown in Fig. 3 (25). Other protein targets include DNA polymerase subunit γ (POLG) and the Twinkle helicase (9). Protein constituents of nucleoids may be present at different levels, depending on the structure and functional state of the mtDNA. For example, POLG induces replication of mtDNA and thereby labels replicating mtDNA preferentially (21), whereas methylation of mtDNA may reduce binding of TFAM (56).

### Fluorescently tagged mtDNA-binding proteins

TFAM tagged with a fluorescent protein is a commonly used marker of mitochondrial nucleoids (17, 21, 33). Potential limitations of this method are that overexpression of exogenous TFAM can increase the number of nucleoids per cell (21) and that fluorescent proteins may be less bright and photostable relative to some fluorescent dyes.

A fluorescently tagged processivity subunit of POLG (POLG2-GFP) can be used to specifically label replicating mtDNA within cells (21, 57) (Fig. 2), and together with the simultaneous labeling of all mtDNA nucleoids, this can reveal subcellular heterogeneity in the replicative status of mtDNA (21, 57). Future advances in this field may include animal models or cell lines with endogenously tagged TFAM, POLG, and other proteins associated with mtDNA nucleoids, whereby overexpression artifacts are avoided.

### In situ hybridization probes

*In situ* hybridization probes can detect mtDNA and have been used in many applications, including the comparison of mtDNA copy numbers in different tissues (6), visualizing the removal of deleterious mtDNA from the germline after mitochondrial fragmentation (12), and visualization of mtDNA association with particular nucleoids and the resulting genetic autonomy of nucleoids (28).

### Labeling specific mtDNA variants in cells

Many of the aforementioned methods for visualizing mtDNA do not reliably distinguish between sequence variants of mtDNA. Successful labeling of specific variants of mtDNA and heteroplasmy within single cells would greatly enhance the understanding of the mechanisms involved in heteroplasmic mitochondrial disease (*e.g.* by helping to resolve whether mtDNA variants are degraded or segregated differentially in cells and during cell division). Progress has been made toward this using several techniques, but each currently has drawbacks that limit their application. Only a single copy of any specific region of an mtDNA allele is present within each strand of the double-stranded mtDNA. This poses a challenge for achieving adequate signal/noise ratios for stoichiometric probes, and it may be advantageous to employ an amplification step to increase the signal/noise ratio. For *in vitro* assays of mtDNA variants, PCR-based amplification is often used, but this is prone to PCR-derived point mutations due to the imperfect fidelity of the DNA polymerases. Duplex sequencing can be used to control for the occurrence of these mutations and as a sensitive method to detect rare sequence variants of mtDNA *in vitro* (58, 59).

Fluorescence *in situ* hybridization can selectively label specific alleles or specific mtDNA variants *in situ*, but often only with partial selectivity that may be dependent on context (12, 28, 60). This is also limited to fixed, permeabilized cells, and the specificity of the hybridization probes is often limited to the differential labeling of mtDNA variants containing large deletions, rather than single-nucleotide variations, and may provide limited sensitivity (60).

Padlock probes (61) can be used to achieve specificity for single-nucleotide variants of mtDNA *in situ* (62). This technique employs a peptide nucleic acid probe to locally open a target DNA site, allowing a “padlock” DNA probe to access the site and become ligated. A rolling circle amplification then generates thousands of single-stranded copies of the target sequence that can be visualized with fluorescent *in situ* hybridization (61, 62). Detection efficiency for padlock probes at target sites can be in the region of 90% (62). This technique is limited to fixed, permeabilized cells and often confers only limited specificity for single-nucleotide variants due to substantial binding of the probe to the other variants (61, 62).

Labeling of mtDNA containing single-nucleotide variations has also been achieved *in situ* using a technique termed CasPLA (63). In this technique, Cas9/guide RNA (gRNA) is directed to a specific sequence on mtDNA, and local amplification of a fluorescence signal is achieved via a proximity ligation assay. This can enable visualization of individual nucleoids containing the specific mtDNA sequence of interest (63). The low copy number of mtDNAs in nucleoids (25) confirms that CasPLA can achieve high sensitivities at the level of single molecules. Fixed, permeabilized cells are required for the method, to enable access of the probes to the mtDNA; there is currently no reported method for visualizing single-nucleotide mtDNA variants within live cells (63).

## Imaging methods for visualizing mtDNA in cells

### Superresolution techniques and tools.

SRM comprises an array of techniques, such as photoactivated localization microscopy (PALM), stochastic optical reconstruction microscopy (STORM), and stimulated emission depletion (STED) microscopy, that overcome the diffraction barrier and can achieve a resolution <10 nm and thereby generate novel biological insights (64–67).

SRM has enabled insights into the structure and dynamics of submitochondrial components, including mtDNA and nucleoids. For example, it has shown that mitochondrial cristae undergo remodeling on a submitochondrial level (68); that the organization of cristae may be spatially and functionally linked to the mitochondrial transport machinery at endoplasmic reticulum–mitochondria contact sites (69); that mitochondrial nucleoids occupy voids between cristae (27); and that individual cristae show remarkable functional independence, including different membrane potentials (68). These techniques achieve resolutions capable of determining the structure and composition of individual nucleoids and nucleoid clusters (24, 25).

SRM entails unique requirements for the fluorescent labels that are used to visualize mtDNA or other structures, as well as suitable cameras and analysis software (67). For example, STORM requires fluorophores, such as Alexa Fluor 647, that alternate between dark and emitting states, or “blink”, upon exposure to illumination. SRM imaging of mtDNA by PALM has been achieved using an overexpressed TFAM tagged with a photoconvertible fluorescent protein, mEos2 (24). SRM imaging of mtDNA has also been achieved with STED using antibodies directed against DNA and TFAM (Fig. 3) (25). Newer red fluorescent DNA-binding dyes that label mtDNA have been described recently and may prove useful for SRM applications (39, 43, 44). Most SRM methods are constrained to imaging only one or two different labels with superresolution in any given sample, but some allow several labels to be imaged at superresolution. These include 4Pi single-molecule switching (4Pi-SMS) microscopy, which is reported to enable imaging of three labels in three dimensions, at 5–10 nm resolution, and has been applied to the imaging of mtDNA (70).

### Other methods for visualizing mtDNA in situ

Light-sheet microscopy can enable rapid and high-resolution imaging of relatively large samples, such as embryos (71, 72), and has been used to visualize the escape of mtDNA from mitochondria during apoptosis (73).

**Figure 4.**
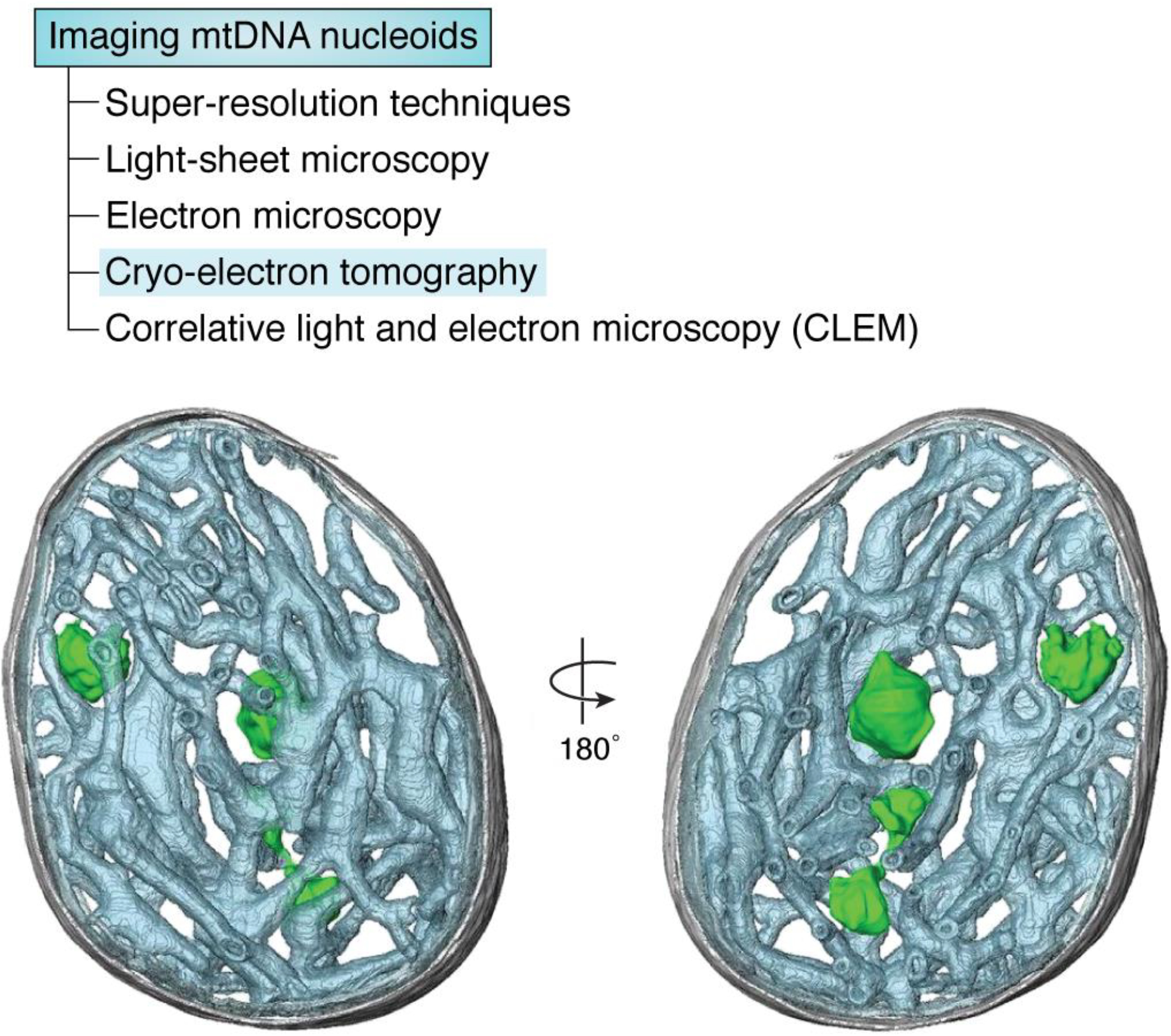
**Mitochondrial nucleoids observed by cryo-electron tomography *in situ*.** A *segmented surface representation* shows the position of mitochondrial nucleoids (*green*) in a bovine heart mitochondrion. *Green*, nucleoids; *gray*, outer membrane; *gray-blue*, cristae. The images are reproduced from previously published data (74), reprinted with permission from the United States National Academy of Sciences.

EM has been used to demonstrate the compaction of mtDNA by TFAM *in vitro* (74) and the mitochondrial herniation of mtDNA *in situ* (73). Visualization of mtDNA by EM may typically be achieved by negative staining and platinum shadowing *in vitro* (74) or immunogold labeling of TFAM *in situ* (73). Electron cryotomography has been used to visualize mtDNA nucleoids at high resolution within isolated mitochondria after rapid freezing (74) (Fig. 4), and this method can obviate the need for chemical fixation, dehydration, heavy metal staining, and sectioning (75). Correlative light and EM images the same sample with both EM and fluorescence microscopy. This technique can visualize mtDNA and correlate it with other structural features within cells labeled with fluorescent probes (73, 76, 77).

Although they are beyond the scope of this review and not currently possible *in situ*, biochemical and sequence-based methods can be used to measure the mtDNA copy number *in vitro*. These include quantitative PCR, duplex sequencing (58, 59, 78), microarrays, and DNA-sequencing read counts (16).

### Quantification of mtDNA images

Images of mtDNA and mitochondria within cells can be analyzed quantitatively using an array of packages (79). The most common methods use plugins for the open-source ImageJ software. For example, the plugins TrackMate (80, 81) and PunctaSpeck (82) can track particles such as mtDNA nucleoids within cells and measure their numbers and intensities over time.

The biological properties of mtDNA nucleoids may affect the choice of analysis tools. The diameter of a single nucleoid is ∼100 nm (23–25), and SRM may therefore be required for adequate resolution. Analysis of experiments involving incorporation and labeling of nucleotide analogues in replicating mtDNA (45, 46, 52) may involve time-lapse images acquired over several hours or days in live cells, because >1 h is required for a single mtDNA genome to replicate completely (83, 84). The fluorescence intensity of labeled mtDNA nucleoids may not be linearly related to the amount of mtDNA because factors including the replicative state and epigenetic modifications of mtDNA may alter the binding of dyes or TFAM (56).

The Mitochondrial Network Analysis (MiNA) plugin can analyze the morphology of mitochondrial networks in 3D stacks, to estimate network volumes and lengths of individual structures (85). The open-source Mitograph has also been used to measure the characteristics of the mitochondrial network in cells (19, 86). Commercial packages, such as Imaris (87) and Volocity (88), have also been used to analyze mitochondrial characteristics, such as movement and network distribution, and can track the intensities of mtDNA nucleoids (17). An array of software also exists for analyzing SRM images, such as the open-source SR-Tesseler software for analysis of two-dimensional localization-based superresolution microscopy data (89).

## Manipulating mtDNA in cells

### Tools and techniques for perturbing mtDNA copy number

The mtDNA copy number varies between cells (6, 90) but is often roughly several hundred to several thousand mtDNAs per mammalian somatic cell (17). Manipulation of the mtDNA copy number can be useful to determine the mechanisms involved in mtDNA replication and turnover, to understand the etiology of disease, and to develop novel therapeutics. The mtDNA copy number is altered in many primary human cancers (18, 91). Lowering the mtDNA copy number of some cancer cells leads to increased susceptibility of these cells to anti-cancer drugs (92), whereas decreasing mtDNA copy number in pancreatic cancer cells leads to autophagy-dependent ferroptotic cell death of these cells (93). The copy number of mtDNA also changes with aging in some human tissues. Decreases have been reported in skeletal muscle (5), blood mononuclear cells (5, 94, 95), and kidney (6), whereas an increase was reported in liver (5) and some controversy remains for other tissues (96).

Depletion of mtDNA within cells can be achieved via diverse methods, including chemically, enzymatically, or via manipulation of regulatory proteins or cellular organelles. Cells in which mtDNA is absent are termed rho0 cells and can be generated by a variety of methods, including treatment of cells with ethidium bromide or targeted nucleases (97–99). Introduction of new mtDNA into rho0 cells results in cybrid cells (100, 101), and this technique can yield insights into the function of mtDNA variants. Techniques for directly manipulating mtDNA within cells are summarized in Table 2.

**Table 2:** **Manipulating mtDNA in cells**

Tool/Technique	Mechanism of action	Notes	References
**Chemical treatments**			
ddC	Inhibition of POLG, mtDNA chain termination	Most potent nucleotide analog tested, cellular toxicity in some contexts	103, 142, 143
Azidothymidine	Inhibition of POLG, mtDNA chain termination	Cellular toxicity in some contexts	103, 142, 143
Dideoxyinosine	Inhibition of POLG, mtDNA chain termination	Cellular toxicity in some contexts	103, 142, 143
Dideoxydidehydrothymidine	Inhibition of POLG, mtDNA chain termination	Cellular toxicity in some contexts	103, 142, 143
Dideoxydidehydrocytidine	Inhibition of POLG, mtDNA chain termination	Cellular toxicity in some contexts	103, 142, 143
TFAM RNAi or knockout	Destabilizes mtDNA	Slow action, temporary, specific	115, 144
TFAM overexpression	Increases mtDNA copy number	Slow action, temporary	144, 145
Twinkle overexpression	Increases mtDNA copy number	Slow action, temporary	145, 146
Twinkle RNAi or knockout	Destabilizes mtDNA	Slow action, temporary, specific	146
Ethidium bromide	Intercalation; prevents replication	Cellular toxicity in some contexts	147
**Targeted nucleases**			
Mito-CRISPR/Cas9	Cuts DNA locally at CRISPR/Cas9-binding site, targeting with guide RNA	Locus-specific, requires PAM sites, requires guide RNA access to mitochondrial matrix	148
Mito-TALEN-FokI	Targets and cuts DNA locally at TALEN-binding site	Locus-specific, no guide RNA required	105
Mito-ZFN-FokI	Targets and cuts DNA locally at ZFN-binding site	Locus-specific, no guide RNA required	111
Mito-PstI	Cuts DNA at CTGCAG	Multiple sites on mtDNA, not targetable	113, 149
Mito-XhoI	Cuts DNA at CTCGAG	Multiple sites on mtDNA, not targetable	113
Mito-ScaI	Cuts DNA at AGTACT	Multiple sites on mtDNA, not targetable	150
Mito-EGFP-EcoRI	Cuts DNA at GAATTC	Multiple sites on mtDNA, not targetable	97
**Other**			
DddA cytidine deaminase-TALE	Base editor targeted to mtDNA sequence via fused TALE	Locus-specific, no guide RNA required, no PAM sites required	128

The antiretroviral drug zalcitabine is a nucleoside analog (ddC) that inhibits the mitochondrial POLG and causes a reduction in mtDNA copy number (102). This reduction in mtDNA by zalcitabine can lead to the death of some cancer cells (93). Other nucleoside analogues have been used and have similar effects to zalcitabine, although they are less potent (103).

Targeted cleavage of mtDNA can lead to its degradation in cells (104), in part via proteins that form part of the mtDNA replication machinery (104). Cleavage and breakdown of mtDNA has been achieved using mitochondrially targeted transcription activator–like effector nucleases (TALENs) (105–110) and zinc finger nucleases (ZFNs) (111, 112).

Targeting of restriction enzymes, such as EcoRI, PstI, or XhoI, to mitochondria can also be used to decrease or eliminate mtDNA in cells (97, 113). These enzymes can be placed under tissue-specific or inducible control and can be used to create cell or animal models and define mechanisms of mtDNA copy number control (113).

Manipulation of mtDNA copy number can also be achieved by alteration of the mtDNA replication machinery, and mutation of the key components of the replication machinery can cause a spectrum of heritable diseases (114). TFAM overexpression increases mtDNA copy number (21), whereas knockdown or deletion of TFAM reduces or eliminates mtDNA (115, 116). Perturbing other components of the mtDNA replication machinery may also be expected to alter mtDNA copy number. These components may include POLG, the helicase Twinkle, topoisomerase, mitochondrial RNA polymerase, RNase H1, mitochondrial ssDNA-binding protein, and mitochondrial ligase III (114).

Mitochondrial network fragmentation is intimately involved in the mechanisms of mitophagy and may also enhance the breakdown of mtDNA (2, 12). Altering the levels of the endogenous regulators of mitochondrial network architecture, including Drp1, Opa1, and Mfn1/2 (11), may therefore also impose changes to the mtDNA copy number.

### Repair and elimination of pathogenic mutant mtDNA

Mutant mtDNA can be inherited or acquired (*e.g.* during aging and cellular redox dyshomeostasis) (117). Mutant mtDNA can be pathogenic, and a therapeutic strategy is to shift mtDNA heteroplasmy toward the WT mtDNA species (3, 4, 118) using mitochondrially targeted ZFNs (119) or TALENs (120). Concerns exist that the gRNA necessary for CRISPR/Cas9 editing may have limited access to the interior of mitochondria (119, 121, 122). Another approach to enhance mitochondrial delivery uses microprojectile transformation of mitochondria with plasmid encoding gRNA, Cas9, and DNA repair template (123).

The most widely used and conventional variants of Cas9, such as the SpCas9 variant from *Streptococcus pyogenes* used for cleavage of DNA and gene editing, require protospacer adjacent motif (PAM) sites with a sequence NGG to be present within the target DNA, to enable binding of the Cas9. This presents an additional barrier to mtDNA cleavage and editing because NGG PAM sites, although numerous within nuclear DNA, are scarce in mtDNA (124). Development of Cas9 variants with a less stringent dependence on specific PAM sites (125–127) may now enable their adoption for mtDNA manipulation.

A recently described CRISPR-free method for precise editing of mtDNA at the level of single nucleotides using a bacterial cytidine deaminase may overcome some of the difficulties associated with the use of CRISPR-based methods (128).

Mitophagy also plays a role in the regulation of pathophysiological mtDNA heteroplasmy. A mouse engineered to express the mitophagy sensor, mito-QC, has proved an invaluable resource to further our understanding of mitophagy, heteroplasmy, and mitochondrial architecture (129). Mito-QC is a GFP-mCherry fluorescent reporter targeted to the outer mitochondrial membrane (129). When mitophagy occurs, the fluorescence of green fluorescent GFP, but not red fluorescent mCherry, is quenched, leading to a change in the measured fluorescence ratio. Another recently described mitophagy sensor is mito-SRAI, which may have improved properties (130).

Modulation of mitophagy may comprise a therapeutic strategy in mitochondrial diseases (131, 132). Mitophagy can be stimulated by drugs such as the antibiotic actinonin (133) or the anti-diabetic metformin (134). Stimulation of mitophagy reverses memory impairment in animal models of Alzheimer's disease (135) and delays age-related morbidities. Caloric restriction may extend lifespan in part by effects on mitophagy (136). Conversely, increased mitophagy may also be involved in mitochondrial diseases (132). Reactive oxygen species may also affect mitophagy and impact many diseases and the aging process (136).

## Future challenges and new approaches

Significant challenges remain to enable improved visualization of mtDNA within cells and to reveal the functions and regulation of mtDNA within cells. Many advances have been made, but the existing tools for labeling mtDNA have limitations (Table 1) and do not fulfill the ideal requirements listed earlier. For example, currently used organic DNA-binding dyes bind to both nuclear DNA and mtDNA, they are spectrally limited to 488-nm excitation light, and they bleach relatively rapidly; current strategies for labeling EdU and BrdU require fixation and permeabilization and the use of toxic azides and copper(I); and antibody labeling requires fixation and permeabilization (Table 1). Currently, the best choice for many applications may be the expression of TFAM tagged with a fluorescent protein. This strategy provides selective labeling of mtDNA nucleoids that is relatively bright and photostable to enable video imaging (Fig. 1), although overexpression of fluorescently tagged TFAM may increase mtDNA copy number (21). Development of new tools may help to overcome the existing limitations. For example, live-cell and time-lapse imaging would benefit from improved nonperturbative and photostable mtDNA labels with a range of spectral characteristics and nontoxic labeling of replicating mtDNA. Useful future approaches may include the fluorescent tagging of native proteins such as TFAM or POLG2 via gene editing (81) to obviate the need for overexpression or the use of fluorescently labeled single-domain antibodies (chromobodies) to bind to target proteins in live cells (137).

Visualization of the mtDNA epigenetics and post-translational modification of nucleoids would enhance our understanding of mtDNA physiology and disease. One example is DNA methylation, which is widespread and functionally important (138). 5-Methylcytosine is common in nuclear DNA but is considered to occur less frequently in mtDNA (56). In contrast, *N*^6^-methyldeoxyadenosine (6mA) methylation is reported to occur at 1,300-fold higher levels in mtDNA relative to nuclear DNA and reduces transcription of mtDNA (56). This characteristic methylation signature of mtDNA may play important physiological roles and may also provide an avenue for selective visualization and manipulation of 6mA-methylated mtDNA. An antibody against 6mA stains mitochondria in normal cells but not rho0 cells (56), suggesting that similar antibodies might identify 6mA-methylated mtDNA selectively. A limitation of the 6mA-antibody is cross-reactivity with RNA containing *N*^6^-methyladenosine and the consequent need for stringent RNase treatment to selectively visualize mtDNA (56).

An exciting avenue for development is the visualization of mtDNA breakdown. Indirect methods such as PCR have been utilized to determine some of the proteins and mechanisms involved (104), but a direct method for visualizing breakdown within live cells has not yet been described. Mitophagy is known to turn over entire mitochondria (129), but where the mtDNA is broken down and disposed of during this process remains unclear. It remains possible that mtDNA is metabolized to some extent within mitochondria, in the absence of mitophagy or preceding it. Mitochondria can contain lysosome-like organelles (139) and vesicles containing cytosolic components (140). Whether these intramitochondrial organelles or other factors within mitochondria play roles in mtDNA metabolism remains to be resolved.

Last, a significant challenge is to develop a technique that is capable of visualizing specific single-nucleotide mtDNA variants within live cells. This may be via techniques analogous to the CasPLA (63) or padlock probes (61, 62) used to label DNA variants in fixed and permeabilized cells or via improvements to fluorescence *in situ* hybridization methods (141). The development of new variants of Cas9 may also assist (125–127). Development of these techniques in live cells would lead to a step change in the understanding of the origin and regulation of mtDNA heteroplasmy in physiology and disease.

In summary, a wide variety of experimental tools and techniques exist to visualize, quantify, and manipulate the properties of mtDNA, but the existing tools have limitations. These may be overcome by future technical advances, to facilitate a greater understanding of the roles and regulation of mtDNA and potential therapeutic interventions.

## Data availability

All data are contained within this article. Images in the figures are taken from previously published data (17, 21, 25, 74); figure panels have not been changed; and the images are covered by a license (http://creativecommons.org/licenses/by/4.0/).
